# Shift work and the risk of cardiovascular disease among workers in cocoa processing company, Tema

**DOI:** 10.1186/s13104-015-1750-3

**Published:** 2015-12-18

**Authors:** Henry Asare-Anane, Adams Abdul-Latif, Emmanuel Kwaku Ofori, Mubarak Abdul-Rahman, Seth D. Amanquah

**Affiliations:** Department of Chemical Pathology, School of Biomedical and Allied Health Sciences (S.B.A.H.S), University of Ghana, P. O. Box 4236, Korle-Bu, Accra, Ghana; K.B.T.H, Korle-Bu, Accra, Ghana; Department of Immunology, School of Biomedical and Allied Health Sciences (S.B.A.H.S), Korle-Bu, Accra, Ghana

**Keywords:** Cardiovascular disease, Shift work, Body mass index, Dyslipidemia, Hypertension

## Abstract

**Objective:**

Shift work has been implicated in cardiovascular disease (CVD), a major cause of death globally. The aim of this study was to evaluate the risk of developing CVD in shift work.

**Design:**

A cross-sectional study involving secondary analysis of shift and non-shift work from an industry in Ghana.

**Participants:**

Two hundred (113 shift and 87 non-shift) consecutive workers who consented were recruited into the study. A structured questionnaire was administered to deduce information on participant’s age, alcohol consumption pattern, smoking habits, history of diabetes, stroke and hypertension.

**Results:**

Shift workers were found to be associated with higher body mass index (26.9 ± 4.6 vs 25.2 ± 3.3, p = 0.013); fasting blood glucose (5.9 ± 1.8 vs 5.3 ± 0.8, p ≤ 0.0001); glycated haemoglobin (4.9 ± 0.9 vs 4.2 ± 0.8, p ≤ 0.0001); high sensitivity C-reactive protein (2.5 ± 1.1 vs 1.8 ± 1.1, p < 0.0001); total cholesterol (5.9 ± 1.3 vs 5.2 ± 1.7, p = 0.002); triglycerides (1.3 ± 0.8 vs 1.1 ± 0.6, p = 0.015) and LDL cholesterol (3.6 ± 0.9 vs 3.2 ± 1.3, p = 0.04) than controls. Shift work however, had no associations with HDL-cholesterol.

**Conclusion:**

It can be concluded that shift work is associated with risk factors of CVD.

**Electronic supplementary material:**

The online version of this article (doi:10.1186/s13104-015-1750-3) contains supplementary material, which is available to authorized users.

## Background

A typical work day in most parts of the world is 8 h of work starting from about 7 a.m. or 8 a.m. However, industrialization and technological advancements have created the need for some businesses and organizations to operate outside standard hours with some operating 24 h each day. About 20 % of workers in Europe are employed on shift work involving night work and extended hours [1]. Extended hours of work are generally accepted to mean working more than 48 h a week [2]. The driving factors of the non-standard working hours typically include commercial competitiveness to increase productivity, increased consumer demands for goods and services and government legislation allowing flexible work policies [1]. However, these benefits do not come without any health and safety risks to the employees. Recent studies however put the greater risk of health effects of shift work on permanent or long-term shift workers. One major effect is the risk of developing cardiovascular disease (CVD). A World Health Organization (WHO) report identifies CVD as accounting for 9.2 % of deaths in the African region in 2001 [3]. Review of autopsy cases in Korle-Bu Teaching Hospital indicated that CVDs constituted about one-fifth of all deaths in Korle-Bu Teaching Hospital, Ghana [4]. A number of pathways linking shift work to CVD and related chronic conditions have been proposed, including circadian rhythm disruption, stress caused by disturbance to normal metabolic and hormonal functions [5] and behaviors, such as smoking and poor diet [6]. These factors may contribute to an increase in body mass index (BMI) [5, 6]. It has also been proposed that shift work might result in stress [7, 8]. Such metabolic disturbances may lead to the clinical expression of a number of co-morbidities including central obesity, hypertension, dyslipidaemia and endothelial dysfunction, all being risk factors for CVD [7]. In Ghana, there is little research information on whether shift workers carry any risk of developing CVDs. Many Ghanaians are involved in shift work in the hospitals, industries and other institutions. It is possible that the health problems of shift work reported in other parts of the world such as Europe can also be found among Ghanaians. This study seeks to provide information on the relationship between shift work and CVD among workers in Ghana and also serve as the basis for primary prevention and control of CVD.

## Aim

To investigate the association between shift work and the risk of CVDs at Cocoa Processing Company (CPC), Tema, Ghana.

## Methods

This study was carried out at Ghana CPC, Tema. This was a cross-sectional study involving a convenient sampling approach. Two hundred (200) consecutive workers after informed consent were recruited into the study. Of those asked, 40 % agreed to be part of the study. The study was approved by the Ethical and Protocol Review Board of the University Ghana Medical School in accordance with the Declaration of Helsinki. Rotating shift workers (113) for at least a year were compared with non-shift workers (87). A structured questionnaire was administered to consented subjects to obtain information on participant’s age, alcohol consumption, smoking habits, history of diabetes, stroke and hypertension. Subjects in this study were all mild smokers (smoked less than five cigarette sticks a day). Alcoholics were defined as participants who consumed alcohol on daily basis. Anthropometric measurements such as height and weight were taken and BMI calculated. Blood pressure was taken using a mercury sphygmomanometer and stethoscope after participants had rested for 15 min. Shift workers with family history of CVDs or pregnant women within 1 year of post partum were excluded from the study. Subjects who admitted to eating during the night shift were excluded from the study.

### Laboratory procedure

Eight milliliters (8 ml) of venous blood sample was collected between 6 a.m. and 8 a.m. for all assays, after participants had fasted for 10–14 h. Four milliliters (4 ml) of the blood sample was transferred into a serum separator tube for the analysis of lipid profile and high sensitive C-reactive protein. Two milliliters (2 ml) was further transferred into ethylene diamine tetra acetic acid (EDTA) containing tube for glycated haemoglobin estimation. The remaining 2 ml was transferred into sodium fluoride containing tube for the estimation of glucose. The resulting sera and plasma after processing were aliquoted into eppendorf tubes and stored at −20 °C until required for use. Fasting blood glucose (FBG), total cholesterol (TC), triglycerides (TG), low density lipoprotein (LDL) and high density lipoprotein cholesterol (HDL), were analyzed using the VITROS system chemistry auto-analyzer (version 250) (Ortho Clinical Diagnostics, Rochester, New Jersey, USA). Coronary risk was computed as the ratio of TC to HDL and has been described by Wilson and friends [9]. Glycated haemoglobin determination was based on a latex agglutination inhibition assay by a kit purchased from (Medsource Ozone Biomedicals Pvt, Ltd, India). High sensitive C-reactive proteins (hs-CRP) were analyzed using a kit purchased from Kamiya Biomedical Company, USA.

### Statistical analysis

Data was entered into Microsoft Office Excel 2010 (Louisville, Kentucky) and analyzed with the statistical package for the social sciences (SPSS) version 20. Continuous data were expressed as mean plus or minus standard deviation (mean ± SD). Categorical data was presented as frequencies with percentages in parenthesis. Unpaired Student *t* test was used to evaluate significant differences between two means. Logistic regression was used to calculate the adjusted odds ratios (OR). Variables with significant associations were assessed through multiple regression analysis to determine their independent contributions. p values less than 0.05 were considered significant.

## Results

A total of 200 volunteers participated in the study. These were made up of one hundred and thirteen (113) rotating shift workers and eighty-seven (87) non-shift workers (control group). The general characteristics of the study population are shown in Table 1. The proportion of subjects who smoked cigarettes or drank alcohol was not statistically significant (p > 0.05) between the two groups. The mean ages for the shift and the non-shift workers were 42.0 and 40.3 years respectively. Difference in age between shift workers and non-shift workers was not statistically significant (p = 0.331). BMI of the shift working group was higher than that for the non-shift working group and was statistically significant (p = 0.013). There were however, no significant differences in age (p = 0.331), SBP (p = 0.076), DBP (p = 0.529) and waist hip ratio (WHR) (p = 0.303) between the shift workers and controls (Table 1). The mean FBG, serum TC, low density lipoproteins, TG and glycated haemoglobin levels were significantly higher in shift workers compared to controls (p < 0.004) (Table 1). Also, the mean hs-CRP was significantly increased in the shift workers group compared to the non-shift workers (p < 0.0001). No significant differences in high density lipoprotein and coronary risk were observed between shift workers and non-shift workers (p > 0.05). Associations between several parameters (TC, TG, HDL, FBG, hs-CRP, HbA1c, BMI and WHR) with shift work were determined and shown in Table 2. Models have been adjusted for known risk factors. OR showed that shift work was intimately and independently related to higher levels of highly sensitive C-reactive proteins (OR = 3.20, p = 0.032). Table 3 shows the association (regression) between BMI and the biochemical risk markers of CVD. The results showed that BMI associated significantly with WHR (β = 0.31, p = 0.001), hs-CRP (β = −0.48, p = 0.018) and coronary risk (β = 0.74, p = 0.001). TC, triglyceride and low density lipoprotein however, associated insignificantly (p > 0.05) with BMI.

**Table 1:** General characteristics of the study population

Parameters	Shift workers (N = 113)	Non-shift workers (N = 87)	p value
Socio-demographic parameters			
Smokers	12 (10.62)	10 (11.49)	0.083
Alcoholics	38 (33.63)	24 (27.59)	0.118
Family history of diabetes	42 (37.17)	35 (40.23)	0.204
Family history of hypertension	37 (32.74)	41 (47.13)	0.061
Clinical parameters			
Age (years)	42.0 ± 8.2	40.3 ± 11.5	0.3310
SBP (mmHg)	122.3 ± 20.3	118.8 ± 20.1	0.0760
DBP (mmHg)	81.8 ± 13.2	81.2 ± 10.7	0.5290
BMI (kg/m^2^)	26.9 ± 4.6	25.2 ± 3.3	0.0130*
WHR (ratio)	0.91 ± 0.12	0.88 ± 0.07	0.3030
Period of shift (years)	13.2 ± 7.8	–	–
Biochemical parameters			
FBG (mmol/L)	5.9 ± 1.8	5.3 ± 0.8	0.0001**
HbA1c (%)	4.9 ± 0.9	4.2 ± 0.8	0.0001**
TC (mmol/L)	5.9 ± 1.3	5.2 ± 1.7	0.0020*
TG (mmol/L)	1.3 ± 0.8	1.1 ± 0.6	0.0015*
HDL (mmol/L)	1.6 ± 0.5	1.5 ± 0.6	0.0970
LDL (mmol/L)	3.6 ± 0.9	3.2 ± 1.3	0.0040*
VLDL (mmol/L)	0.6 ± 0.4	0.5 ± 0.3	0.0140*
Coronary risk (ratio)	4.0 ± 1.0	3.8 ± 1.1	0.3770
hs-CRP (U/L)	2.5 ± 1.1	1.8 ± 1.1	0.0001**

The table shows the results for the general characteristics of the study population

Continuous data were presented as mean ± standard deviation

Categorical data are presented as frequencies with percentages in parenthesis

*HbA1c* glycated haemoglobin, *hs*-*CRP* highly sensitive C-reactive protein, *TC* total cholesterol, *TG* triglyceride, *HDL* high density lipoprotein, *LDL* low density lipoprotein, *VLDL* very low density lipoproteins, *SBP* systolic blood pressure, *DBP* diastolic blood pressure, *WHR* waist hip ratio, *FBG* fasting blood glucose, *BMI* body mass index

* Mean difference is significant (p < 0.05)

** Mean difference is highly significant (p < 0.0001)

**Table 2:** Risk ratio of the modifiable risk factors of cardiovascular diseases in shift work

Risk factors	Adjusted odds ratio	95 % CI	p value
TC	1.90	0.93–2.24	0.6545
TG	2.45	0.8–4.70	0.4316
HDL	0.40	0.13–0.73	0.016**
FBG	1.26	0.59–2.34	0.6736
hs-CRP	3.20	1.31–4.76	0.0318*
HbA1c	2.70	0.9–8.42	0.1899
WHR	0.54	0.50–0.90	0.0008**
BMI	1.32	0.67–1.07	0.1096

The table shows the association between several parameters (TC, TG, HDL, FBG, hs-CRP, HbA1c, BMI and WHR) with shift work

*HbA1c* glycated haemoglobin, *hs*-*CRP* highly sensitive C-reactive protein, *TC* total cholesterol, *TG* triglyceride, *HDL* high density lipoprotein, *WHR* waist hip ratio, *FBG* fasting blood glucose, *BMI* body mass index, *OR* adjusted odds ratio

* Mean difference is significant (p < 0.05)

** Mean difference is highly significant (p < 0.0001)

**Table 3:** Multiple regression analysis of several correlates with BMI

Variables	Coefficient	95 % CI		p value
WHR (ratio)	0.31	6.17	21.56	0.001**
TC (mmol/L)	0.24	−0.17	1.27	0.136
TG (mmol/L)	0.09	−0.53	1.25	0.426
LDL (mmol/L)	−0.11	−1.51	0.72	0.365
hs-CRP (U/L)	−0.48	−2.71	0.26	0.018*
Coronary risk	0.74	1.07	3.73	0.001**

The analysis used BMI as an independent variable and several parameters (TC, TG, hs-CRP, coronary risk and WHR) as the dependent variables

*hs*-*CRP* highly sensitive C-reactive protein, *TC* total cholesterol, *TG* triglyceride, *WHR* waist hip ratio, *LDL* low density lipoprotein

* Mean difference is significant (p < 0.05)

** Mean difference is highly significant (p < 0.0001)

Table 4 shows the Framingham Study risk score for both shift and non-shift workers. The Framingham score is a cardiovascular risk score model for predicting the risk of developing CVDs in the next 10 years. Results showed that 79 % of the shift workers and 84 % of the non-shift workers had low risk of developing CVDs respectively. There were no workers with high risk of developing CVD in this study.

**Table 4:** The Framingham Study risk score for shift and non-shift workers

Risk score	% of shift workers	% of non-shift workers
Low risk	79	84
Moderate risk	21	16
High risk	0	0
Total	100 %	100 %

The Framingham score is a cardiovascular risk score model for predicting the risk of developing cardiovascular diseases in the next 10 years. The table shows the percentage of workers in each risk score class. The Framingham score is reported in percentage terms

**Fig. 1:**
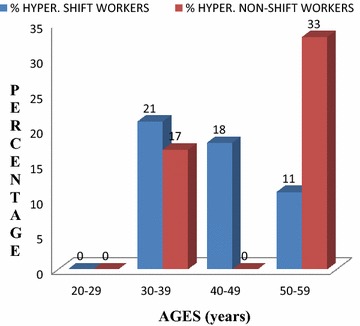
Age distribution of hypertensive shift and non-shift workers

Figure 1 shows the prevalence of hypertension among the study subjects. Hypertension was more prevalent among shift workers aged between 30 and 49 years.

## Discussion

Shift working has become one of the most popular work schedules employed to effectively utilize the relatively few employees to carry out round the clock activities. This exposes the employees to working under pressure, night wakefulness, smoking, poor dieting, sleeping and waking at odd times [6]. These life styles lead to disturbance in normal metabolic and hormonal functions of the body which subsequently result in stress and circadian rhythm disruption [6]. This study evaluated some clinical and biochemical markers for the development of CVDs among shift and non-shift workers. In this study, the mean BMI of shift workers was significantly higher than that of the non-shift workers. This result is consistent with earlier findings [10–14]. The high BMI associated with shift workers may be due to adoption of unhealthy lifestyles such as eating at night during night shift work which has been reported to expose people to weight gain [15]. In this study also, BMI was seen to predict WHR, hs-CRP and coronary risk (Table 3). BMI and WHR have been reported in predicting the risk of developing type 2 diabetes [16, 17]. Elevated BMI is a well-established contributor to the etiology of CVDs [18, 19]. The study revealed no significant differences between the mean SBP and DBP for shift and non-shift workers. This was at variance with the observation made by others [20–22]. The reasons for these observations in this study is not clear, but could be attributed to the use of cocoa drink products by the workers and the different study populations used. When categorized by age, Fig. 1 showed that hypertension was more prevalent among shift workers aged between 30 and 49 years. This could be attributed to job related stress. Many of the workers in this category admitted that the low salary levels compelled them to engage in overtime duties to enhance their earnings. Stress is a predominant factor in the risk of developing hypertension [22]. This study also showed a decreasing trend in the prevalence of hypertension among the shift workers aged 50–59 years. This observation could be due to more stressful jobs being given to the younger ones and leaving the less stressful jobs for the older workers. Continued shift work could also lead to adaptation to biological rhythms that do not promote hypertension. Continual consumption of cocoa products may have also reduced blood pressure and lowered the risk of CVDs [23, 24]. This study associated raised FBG and HbA1c with shift work. This can be attributed to the reduced sleep period common among the shift workers, leading to impaired glucose metabolism as reported by others [25, 26]. Increased FBG will lead to non-enzymatic attachment of glucose to haemoglobin to eventually form glycated haemoglobin. Increased glycation will lead to the formation of advanced glycosylated end products, which induces the production and secretion of inflammatory cytokines such as CRP.

The mean values for TC, TG, low density lipoproteins (LDL) and very low density lipoproteins (VLDL) were significantly higher among the shift workers compared to controls (Table 1). These results agreed with prior studies on elevated cholesterol levels among shift workers [21] and on elevated TG associated with shift work [5, 26, 27]. This may be due to high incidence of reduced sleep duration associated with the shift workers. Reduced sleep has been linked to disruption of biological rhythms and hence dyslipidaemia [28]. An alternative explanation could be that the subjects may have eaten during the night shift. This is however a remote possibility, since subjects who admitted to eating during night shift were disqualified from the study.

This study associated shift work with high risk for CVD using hs-CRP as the risk marker (Table 2). This result concurs with earlier reports [29, 30]. CRP is highly stable and allows for accurate measurements without special collection procedures [31]. CRP is an independent predictor that adds information to lipid screening and to the Framingham scores [31, 32]. CRP increases plasminogen activator inhibitor-1 (PAI-1) expression and activity. Increase PAI-1 indicates lowered plasminolysis and thus leads to atherogenesis [32]. CRP can also localize atherosclerotic lesions by promoting complement activation. Activated complement may provoke vasoconstriction of coronary vessels [33].

The Framingham risk score is a scoring system that gives an estimate of the probability of a person developing CVD in the next 10 years. The scoring system takes into consideration factors such as age, sex, LDL Cholesterol, HDL Cholesterol, blood pressure (and whether the person is treated or not for hypertension), diabetes and smoking. It is an online calculator which incorporates all these risk factors. People with low risk have 10 % or less CVD risk, intermediate risk have 10–20 % CVD risk and high risk 20 % or more CVD risk [9]. The Framingham Study risk score (Table 4) shows that shift workers in CPC, do not carry high risk of developing CVD. This could be due to the fact that there were generally low levels of the biochemical risk markers found in the blood of the workers, attributable to the consumption of the cocoa products by the factory workers [23, 24]. The factory floor workers have direct access to the cocoa products they manufacture and could be benefiting from the health benefits of cocoa products. Again, the low risk score can also be attributed to the low prevalence of smoking and alcoholism among the shift workers of CPC. Again, it could be that shift workers carrying out active work indulge in regular exercises which could have also been beneficial, hence the negative predictive value.

## Conclusion

Data from this study has shown that workers of CPC in Tema who do rotational shift work were associated with high BMI, FBG, glycated haemoglobin, hs-CRP and dyslipidaemia. This study showed that high sensitivity C-reactive protein was the most effective marker for the assessment of CVD in workers of CPC. There were no associations with HDL-cholesterol, waist-hip ratio, systolic and diastolic blood pressure. Hypertension was more prevalent among shift workers aged 30–49 years. It can be concluded that shift work is associated with risk factors of CVDs.

## Limitations

This study however could not establish whether the consumption of the cocoa products by the workers had a direct effect on reducing their risk of developing CVDs. Further work should be done in a larger population to ascertain this. Potential confounders such as stress and the quality of sleep were not considered in this study (Additional files 1, 2).

## Additional files

10.1186/s13104-015-1750-3 Questionnaire.
10.1186/s13104-015-1750-3 Strobe checklist.

## Abbreviations

BMI: body mass index
CVD: cardiovascular disease
CPC: cocoa processing company
EDTA: ethylene diamine tetra acetic acid
FBG: fasting blood glucose
hs-CRP: high sensitivity C-reactive protein
TG: triglycerides
TC: total cholesterol
OR: odds ratio
WHR: waist hip ratio
