# Supplementary material for: Shift work and the risk of cardiovascular disease among workers in cocoa processing company, Tema
Source: BMC Res Notes. 2015 Dec 18;8:798. doi: 10.1186/s13104-015-1750-3 (PMC4683766; doi:10.1186/s13104-015-1750-3)
Supplement: Supplementary file 1 — 10.1186/s13104-015-1750-3 Questionnaire. [file 13104_2015_1750_MOESM1_ESM.docx]

**QUESTIONNAIRE**

**FORM S SHIFT GROUP ID: .SG......................................**

The benefits of the study are to educate you on how your lifestyle affects your health and also to ascertain the health burden of cardiovascular diseases (as a result of shift work) on the community. Participation is entirely voluntary and strictly confidential. You may choose to withdraw from the study whenever you wish.

Please provide us with the following information and answer the questions to the best of your ability.

Weight ...........kg Height ...............cm

Waist circumference ..........cm Hip circumference ...........cm

Systolic BP ...........mmHg Diastolic BP ...........mmHg

1. Age…….
2. Marital status a. single b. married c. divorced d. co-habitating
3. Gender: Male Female
4. What is your highest level of education? a No formal education b. Primary school c. Junior High School d. Technical school e. Senior high f. Tertiary
5. What is your current position on the job ................................
6. Which level are you in your current employment

a. Operations b. Supervisor c. Middle Management d. Senior Management

1. Which year did you begin working? ………………………
2. Have you had major breaks during employment aside annual leave?Yes No
3. If yes what is the period of the break …………………………………………
4. Are you currently engaged in shift work? Yes No
5. If yes, what is the period of your shift work? Please select the one(s) that suits you. You can select more than one. For each one you select please indicate the time period of work against it.

a. Morning to Afternoon, from .......... to .........

b. Afternoon to evening, from ........ to ...

c. Evening to Night, from ......... to ...........

d. Evening to Morning, from ........ to .............

e. Night to morning, from .......... to ..........

1. How many years have you been running shift work? ...................years
2. How many years have you been on the **night** shift .............years
3. Have you had some breaks from the night shift work since you started? Yes No
4. How long was the break …………….............................................................
5. How many days of the shift work do you do during the week
6. a. 1 b. two c. three d. four e. five f. six g. seven
7. How many days of the shift work do you do during the month……………………
8. Do you rest or sleep partway during the shift? Yes. No.
9. How long do you usually rest or sleep during the **night** working period
   1. Less than 30 minute b. 30 min to 1 hour c. 1 to 2 hours d. 2 to 3 hours e. 3 to 4 hours f. 4 to 5 hours g. More than 5 hours
10. Do you rest or sleep with the lights on at work? Yes No
11. Do you eat during the night shift period? Yes No
12. What time(s) do you eat in the evening at home? .............................................................
13. What time(s) do you eat in the night on shift work?
14. What type of food do you usually take ……………………………………………………
15. Do you sleep during the day, after the night shift? Yes No
16. How many hours do you sleep during the day after night shift? …………hours
17. Do you sleep in a dark room at home? Yes No
18. Do you job during the day after night shift? Yes No
19. How many hours of job do you do? .......................hours
20. Are you a current smoker? Yes No
21. If yes, how many sticks do you smoke in a day a. 1 b. 2 c. 3 d. 4 e. 5 f. More than 5
22. If no are you a past smoker? Yes No
23. If yes, when did you stop smoking? ...............................
24. Do you take alcohol? Yes No
25. If yes how many bottles do you take in a week? …………..
26. If no did you drink in the past? Yes No
27. Which year did you stop drinking? ……………………….
28. Do you take coffee to stay awake for the night work? Yes No
29. How many times in a week do you often take coffee? ......................
30. Do you take energy drink to stay awake for the job? Yes No
31. Does your work keep you sitting at one place for long? Yes No
32. How long do you sit at work? ..................................
33. How often do you get up from your seat and walk around? ............
34. Do you engage in any regular exercises at home? Yes No
35. If yes how many minutes do you exercise in a day ………………….
36. How many times do you exercise in a week ..................................
37. Which year did you start doing exercises .................................
38. Are you on any cholesterol lowering medication? Yes No
39. Are you on anti-hypertensive medication? Yes No
40. Are you on any diabetes medication Yes No
41. Are you diabetic? Yes No
42. Do you have a close family history of hypertension? Yes No
43. Do you have a close family history of diabetes Yes No
44. Do you have a family history of stroke? Yes No
