# Supplementary material for: Shift work and the risk of cardiovascular disease among workers in cocoa processing company, Tema
Source: BMC Res Notes. 2015 Dec 18;8:798. doi: 10.1186/s13104-015-1750-3 (PMC4683766; doi:10.1186/s13104-015-1750-3)
Supplement: Supplementary file 2 — 10.1186/s13104-015-1750-3 Strobe checklist. [file 13104_2015_1750_MOESM2_ESM.doc]

STROBE Statement—Checklist of items that should be included in reports of ***cross-sectional studies***

|  | Item No | Recommendation |
| --- | --- | --- |
| **Title and abstract** | 1 | (*a*) Shift work and the risk of cardiovascular disease among workers in cocoa processing company, Tema |
|  |  | (*b*) See manuscript |
| Introduction | | |
| Background/rationale | 2 | Shift work has been implicated in cardiovascular disease, a major cause of death globally. In Ghana, there is little research information on whether shift workers carry any risk of developing cardiovascular diseases. |
| Objectives | 3 | To investigate the association between shift work and the risk of cardiovascular diseases at Cocoa Processing Company, Tema, Ghana |
| Methods | | |
| Study design | 4 | A cross-sectional study involving secondary analysis of shift and non-shift work from an industry in Ghana |
| Setting | 5 | The study was carried out at cocoa processing company, Tema, Ghana. |
| Participants | 6 | (*a*) Forty percent (40%) of those asked, agreed to be part of the study. Two hundred consecutive workers who volunteered after written consent were recruited into the study. Subjects who admitted to eating during the night shift were excluded from the study. |
| Variables | 7 | All variables have been listed in the methods and results section of the manuscript |
| Data sources/ measurement | 8* | Data was entered into Microsoft Office Excel 2010 (Louisville, Kentucky) and analyzed with the Statistical Package for the Social Sciences (SPSS) version 20 |
| Bias | 9 | Parameters were adjusted to remove bias |
| Study size | 10 | Minimum study size was obtained by means of a formula |
| Quantitative variables | 11 | All variables have been listed in the methods and results section of the manuscript. Framingham score for predicting the risk of developing cardiovascular diseases in the next 10 years was described and used in this study. Coronary risk was computed as a ratio of total cholesterol to high density lipoprotein. Body mass index was computed as weight in kilograms per squared height in meters. |
| Statistical methods | 12 | (*a*) Continuous data were expressed as mean plus or minus standard deviation (means ± SD). Categorical data was presented as frequencies with percentages in parenthesis. Unpaired student t-test was used to evaluate significant differences between two means. Logistic regression was used to calculate the adjusted odds ratios. Variables with significant associations were assessed through multiple regression analysis to determine their independent contributions. P-values less than 0.05 were considered significant. |
| Results | | |
| Participants | 13* | (a) A total of 200 volunteers participated in the study. Details have been provided in manuscript. |
|  |  | (b) N/A |
|  |  | (c) N/A |
| Descriptive data | 14* | (a) Characteristics of study participants have been provided in the results section of the manuscript. |
|  |  | (b) There were no missing data on participants |
| Outcome data | 15* | See the Framingham risk score in table 4 |
| Main results | 16 | (*a*) See results section of manuscript. Models were adjusted for all known risk factors of cardiovascular disease on which information was collected including body mass index, blood pressure, fasting lipids and sugar, history of hypertension, smoking and diabetes mellitus. |
| Other analyses | 17 | N/A |
| Discussion | | |
| Key results | 18 | This study associated shift work with high risk for cardiovascular disease using hs-CRP as the risk marker (table 2).The Framingham Study risk score showed that shift workers in cocoa processing company did not carry high risk of developing cardiovascular disease (table 4). This could be attributable to the consumption of the cocoa products by the factory workers. |
| Limitations | 19 | This study however could not establish whether the consumption of the cocoa products by the workers had a direct effect on reducing their risk of developing cardiovascular diseases. |
| Generalisability | 21 | Since this study did not primarily consider the influence of ethnicity on cardiovascular disease and also, which cocoa product specifically contributed to lowering cardiovascular risk, generalisations remain unclear. |
| Other information | | |
| Funding | 22 | None. |

*Give information separately for exposed and unexposed groups.

**Note:** An Explanation and Elaboration article discusses each checklist item and gives methodological background and published examples of transparent reporting. The STROBE checklist is best used in conjunction with this article (freely available on the Web sites of PLoS Medicine at http://www.plosmedicine.org/, Annals of Internal Medicine at http://www.annals.org/, and Epidemiology at http://www.epidem.com/). Information on the STROBE Initiative is available at www.strobe-statement.org.
